# The Impact of Cardiac Devices on Patients’ Quality of Life—A Systematic Review and Meta-Analysis

**DOI:** 10.3390/jcdd9080257

**Published:** 2022-08-10

**Authors:** Kevin Willy, Christian Ellermann, Florian Reinke, Benjamin Rath, Julian Wolfes, Lars Eckardt, Florian Doldi, Felix K. Wegner, Julia Köbe, Nexhmedin Morina

**Affiliations:** 1 Department for Cardiology II: Electrophysiology, University Hospital Münster, 48149 Münster, Germany; 2 Department of Psychology, University of Münster, 48149 Münster, Germany

**Keywords:** review, meta-analysis, quality of life, cardiac device

## Abstract

The implantation of cardiac devices significantly reduces morbidity and mortality in patients with cardiac arrhythmias. Arrhythmias as well as therapy delivered by the device may impact quality of life of patients concerned considerably. Therefore we aimed at conducting a systematic search and meta-analysis of trials examining the impact of the implantation of cardiac devices, namely implantable cardioverter-defibrillators (ICD), pacemakers and left-ventricular assist devices (LVAD) on quality of life. After pre-registering the trial with the PROSPERO database, we searched Medline, PsycINFO, Web of Science and the Cochrane databases for relevant publications. Study quality was assessed by two independent reviewers using standardized protocols. A total of 37 trials met our inclusion criteria. Of these, 31 trials were cohort trials while 6 trials used a randomized controlled design. We found large pre-post effect sizes for positive associations between quality of life and all types of devices. The effect sizes for LVAD, pacemaker and ICD patients were g = 1.64, g = 1.32 and g = 0.64, respectively. There was a lack of trials examining the effect of implantation on quality of life relative to control conditions. Trials assessing quality of life in patients with cardiac devices are still scarce. Yet, the existing data suggest beneficial effects of cardiac devices on quality of life. We recommend that clinical trials on cardiac devices routinely assess quality of life or other parameters of psychological well-being as a decisive study endpoint. Furthermore, improvements in psychological well-being should influence decisions about implantations of cardiac devices and be part of patient education and may impact shared decision-making.

## 1. Introduction

The implantation of a cardiac device in patients with arrhythmias and in patients with heart failure is associated with relevant survival benefit and improvement of cardiopulmonary exercise capacity in dependence of the type of device. Due to technical progress in the last two decades, the variety of options for implantable cardiac devices for many different clinical situations and indications is diverse [1]. They reach from pacemaker implantations for bradycardia and consecutive asymptomatic ventricular stimulation over implantable cardioverter-defibrillator (ICDs) aimed at terminating life-threatening ventricular arrhythmias with shock deliveries to heart assist devices (LVADs) completely taking over cardiac functions [2].

Importantly, the implantation of the device in the context of life-threatening arrhythmias or survived sudden cardiac death is often accompanied by severe psychological distress. In addition, patients receiving a device for primary prevention are confronted with the need to implant a device into their body that influences their heart function and rhythm [3]. Furthermore, cardiac device therapy is afflicted with long-term complications such as failure of intracardiac leads or infections [4,5]. To that effect, cardiac device therapy is likely to influence the quality of life and other facets of psychological well-being, which in practice may often be underappreciated, underdiagnosed and undertreated [6,7,8]. Several trials have examined the impact of cardiac device therapy on quality of life [9,10]. However, we still lack a systematic review that quantitatively summarizes this literature. Against this background, we aimed at providing a quantitative summery of the existing literature on the impact of cardiac device therapy on quality of life.

## 2. Methods

The aims and the methodology of this meta-analysis were preregistered with the PROSPERO database (https://www.crd.york.ac.uk/PROSPERO/display_record.php?RecordID=233731 accessed on 19 July 2022). At least two independent authors were involved throughout the whole development of this work (research question, searching criteria, quality coding) according to the PRISMA guidelines [11,12].

### 2.1. Search Strategy and Selection of Studies

To be considered eligible, trials had to meet the following inclusion criteria. The trial had to have at least one study arm with a newly implanted cardiac device and report pre/post implantation data. Study participants had to be older than 17 years at the time of implantation. In the final analysis, at least 20 participants in the respective group had to be included and follow-up data after implantation had to be available for at least three months. Publications in English or German language were suitable for inclusion.

After careful investigation of possible MeSH terms and alternative terms, we conducted a literature search in the databases Medline, PsycINFO, Web of Science and the Cochrane library on 28 January 2021 and used the following search strategy and terms: (TI Artificial heart OR AB Artificial Heart OR SU Artificial Heart) OR (TI Protective Devices OR AB protective Devices OR SU Protective Devices) OR (TI defibrillator OR AB defibrillator OR SU defibrillator) OR (TI pacemaker OR AB pacemaker OR SU pacemaker) OR (TI pacing OR AB pacing OR SU pacing) OR (TI left ventricular assist device OR AB left ventricular assist device OR SU left ventricular assist device) OR (TI Implantable loop recorder OR AB implantable loop recorder OR SU implantable loop recorder) OR (TI Heart assist device AND SU Heart assist device AND AB Heart assist device) AND (TI Quality of Life OR AB Quality of Life OR SU Quality of Life) OR (TI Life Satisfaction OR AB Life satisfaction AND SU Life satisfaction) OR (TI satisfaction with life OR SU satisfaction with life OR AB satisfaction with life). The search resulted in 3937 hits.

We inspected titles and abstracts of all hits thoroughly and excluded those that did not meet our inclusion criteria. Thereafter, we read the full texts of all remaining articles by applying our inclusion criteria. Furthermore study populations were checked for double publications. In case of multiple publications using the same study population, we only included the one with the largest sample of patients.

### 2.2. Quality Assessment

All eligible studies were quality coded by two authors (KW and CE) independently. For case control studies we used the Modified Newcastle-Ottawa Quality Assessment Scale (NOS), for cohort studies we used the version of the NOS for cohort studies, which are recommended by the Cochrane Collaboration and for randomized controlled trials we used the Jadad scale, which is the most common instrument used for this purpose [13,14,15].

### 2.3. Coding of Treatment Characteristics

We subdivided the analysis according to two different aspects. First, groups were built because of the underlying device implanted. Therefore, we shared the included papers into three different groups (pacemaker, ICD, LVAD) by means of the implanted device. Second, the publications were classified with respect to the use of an uncontrolled design (i.e., no control group was available and hence treatment efficacy was based on pre- vs. post-assessments) or a controlled design (i.e., treatment efficacy was based on the comparison of an experimental group with a control group). If more than one outcome parameter of quality-of-life was reported, we prioritized the questionnaire expressing subscores for physical and mental aspects of quality of life. For each publication, we extracted the comparison groups, the number of follow-ups and total duration of follow-ups, group sample sizes at follow-up after 6 months and final follow-up, the country the trial was conducted in, the percentage of male and females in the total sample, the mean age, the family status, the year of publication, the statistical analysis used and the total quality score.

### 2.4. Statistical Analysis

Given the limited number of trials meeting our inclusion criteria, especially concerning controlled trials, we decided to first compute within group effect sizes for the impact of treatment on quality of life. Furthermore, a total of 7 trials provided between group analyses. Five of these compared patients with a newly implanted ICD to a control group with unchanged treatment or sole optimization of heart failure therapy. Given recommendations to apply a minimum of four studies for substantial meta-analytic analyses [16], we could not calculate controlled effect sizes. To calculate an effect size, the control group mean was subtracted from the treatment group mean at posttreatment or follow-up, respectively, and divided by the pooled standard deviation.

To obtain the effect size Hedges’ g, the outcome was multiplied by a sample size correction factor *J* = 1 − (3/(4df − 1)) [17]. Analyses were completed with comprehensive meta-analysis (CMA; version 3) [18]. Furthermore, we used random effects model to calculate effect sizes given the heterogeneity of the studies. As a test of homogeneity of effect sizes, we calculated the Q-statistic and the I^2^-statistic that is an indicator of heterogeneity in percentages, with higher percentages indicating high heterogeneity. We chose to interpret Hedges’ g conservatively with Cohen’s convention of small (0.2), medium (0.5) and large (0.8) effects [19]. We considered a *p*-value of 0.05 statistically significant.

If outliers were present, we repeated the respective analysis calculating effect sizes without outliers. An outlier was defined as an effect size at least 3.3 standard deviations below or above the pooled mean [20,21]. Potential predictors were evaluated by the change in Cochrane’s heterogeneity Q-statistic and its associated *p*-value.

## 3. Results

**Figure 1:**
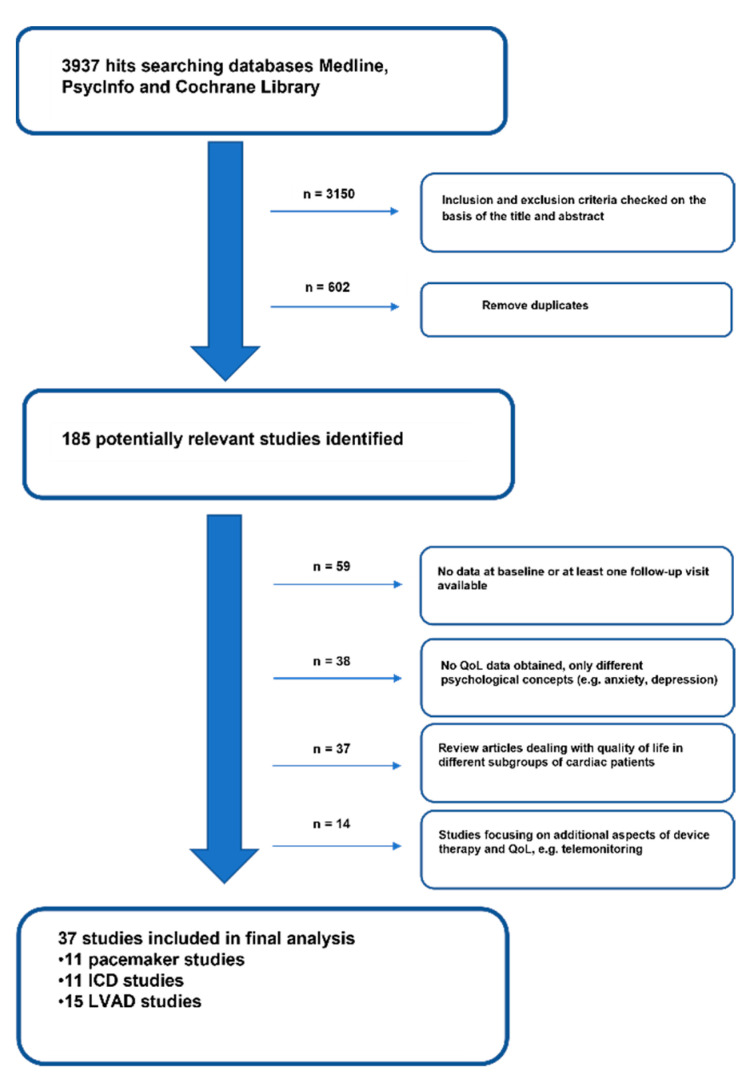
Process of study selection after literature search.

We identified a total of 3937 hits. Figure 1 presents the selection process of potentially eligible trials. After thorough review and further contact with the corresponding authors in order to obtain raw data not included in the respective manuscript, we finally included 37 studies in this meta-analysis.

Of the 37 included studies, 11 trials dealt with patients with newly implanted pacemaker [22,23,24,25,26,27,28,29,30,31,32], 11 trials analyzed patients with new ICDs [33,34,35,36,37,38,39,40,41,42,43] and 15 trials examined patients with new LVADs [44,45,46,47,48,49,50,51,52,53,54,55,56,57,58]. In total, 7 of the 39 trials had control groups not receiving the respective devices while all other trials were cohort studies with longitudinal data acquisition of patients before and after implantation. These trials included 5 publications from the ICD group. Concerning patients with pacemaker or ICDs, most studies used the SF-36 to assess quality of life (17/22) [59]. In studies reporting on patients with LVAD the European Quality of Life-5 Dimension-Visual Analogue Scale (EQ-5D-VAS) [60] and the Kansas City Cardiomyopathy Questionaire on Quality of Life (KCCQ-QoL) [61] was predominantly used (12/15).

**Figure 2:**
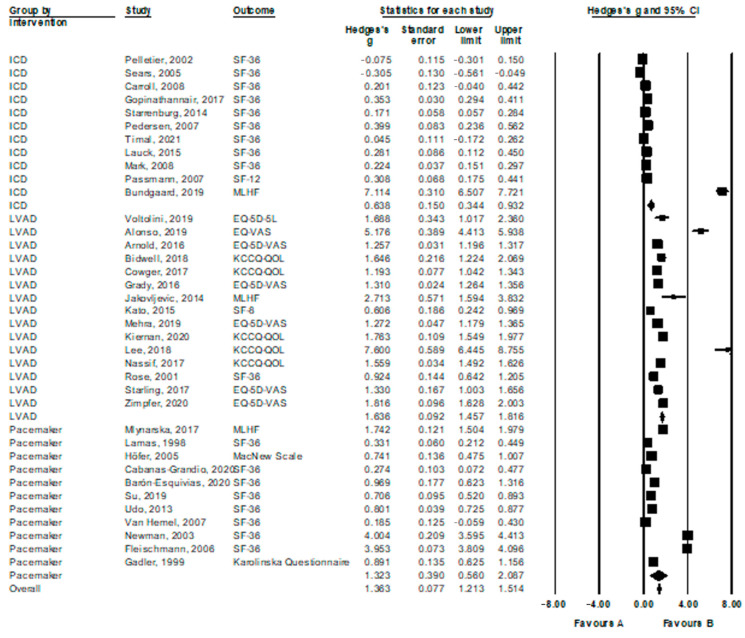
Forest plot of uncontrolled effect size estimates (pre- vs. 6 months follow-up) for the efficacy of device implantation on quality of life.

Generic quality of life: Across all active interventions (k = 37), a large pre-post effect size for the impact of treatment on quality of life was found, g = 1.36, 95% CI = [1.21; 1.51], see Figure 2 for a forest plot. The effect sizes differed, however, across the types of intervention. Both LVAD and pacemaker interventions produced large effect sizes, with g = 1.64, 95% CI = [1.46; 1.82], and g = 1.32, 95% CI = [0.56; 2.09], respectively. ICD interventions on the other hand, produced a medium to large effect size, with g = 0.64, 95% CI = [0.34; 0.93]. Importantly, one trial on ICD interventions [25] and two trials on LVAD interventions [26,27] proved to produce very large effect sizes that we defined as outliers. When conducting the analyses without these outliers, the effect size of ICD interventions was reduced to g = 0.19, 95% CI = [0.09; 0.29], representing a small effect size, whereas the effect size of LVAD interventions remained large, with g = 1.36, 95% CI = [1.25; 1.50].

We further performed subanalyses for physical as well as for mental quality of life, respectively, if the given quality of life assessment allowed differentiation between these two dimensions of quality of life, as for example the SF-36. These sub-analyses were possible for patients with pacemakers and those with ICDs, yet not for patients with LVAD. For both device types, there were 10 studies to be included in the analysis. We found a significant positive association of implantation with physical quality of life in pacemaker (Z-value 39.84) as well as ICD recipients (Z-value 13.30) while the association was stronger for pacemaker patients. Comparable effects were found for psychological aspects of quality of life with an association more strongly exhibited in pacemaker (Z-value 34.21) than in ICD patients (9.25).

Given the low number of trials reporting on potential moderators in the specific type of intervention (i.e., ICD, LVAD and pacemaker), only the following three moderator analyses were possible. Female sex in the LVAD condition explained some heterogeneity (Q = 3.30, *p* = 0.07), indicating that female sex is associated with worse health status as compared to males. Average age in the LVAD group did not significantly account for any heterogeneity. In the ICD group, however, age did explain some heterogeneity (Q = 3.05, *p* = 0.08), which indicated that younger age is associated with better health status.

## 4. Discussion

This meta-analysis revealed a significant improvement of quality of life associated with implantable cardiac devices. The effects were large for pacemaker and LVAD patients, yet they were smaller for patients with ICD.

The efficacy of LVAD on quality of life is likely explained by the very high symptom burden patients implanted with a LVAD suffer from before implantation. Assist device taking over cardiac pump function efficiently relieves symptoms, despite the fact that the implantation process is dangerous and strenuous. Accordingly, the positive effect of pacemakers might be explained by an improvement in physical symptomatology following implantation as the reason for implantation is either a manifest symptomatic bradycardia leading to syncope or symptoms of chronotropic incompetence or enabling a rhythm control therapy in patients with symptomatic supraventricular tachycardia. The only device not leading to immediate improvement of physical symptomatology is the ICD, which is rather a preventive measure to protect the patient from sudden cardiac death. This likely explains why the ICD was associated with fewer improvement in quality of life than in the other two implantable devices.

To our knowledge, this is the first analysis focusing on quality of life in different types of cardiac devices as main outcome parameter. As quality of life is a crucial parameter that may be assessed in every patient, this information is of great value. This is reflected in the increasing appreciation of quality of life parameters in all kinds of different diseases and the growing supporting evidence in this regard over the last years [28,29]. In patients with oncological diseases, authors have recommended regular assessment of quality of life to evaluate the adequacy of diagnostic and therapeutic decisions [28]. The same is true for cardiac patients. Chernoff et al. reported in a meta-analysis that quality of life is significantly influenced by cognitive-behavioral therapy in addition to medical heart therapy [30]. However, a first effort to prove the effect of psychotherapy in patients with cardiac devices (ICD patients in this case) failed as the WEBCARE trial showed no relevant improvement of anxiety, depression and HRQOL in ICD patients who received a 6-lesson web-based cognitive behavioral psychotherapy intervention [31]. A mediator identified in a later sub-analysis was optimism, which was associated with improved mental health as an effect of the intervention [32]. Sobczak-Kaleta et al. reported a large effect of only four lessons of CBT in ICD recipients on quality of life and illness acceptance [33]. However, data on psychological interventions on patients with cardiac devices are still sparse, despite some findings showing that a structured cardiac rehabilitation program and telephone follow-ups answering the patients’ upcoming questions and reducing uncertainty significantly improved psychological adjustment to illness as well as body image concerns after device implantation [34]. These findings support the need for further research in terms of structured rehabilitative and psychological programs and interventions for patients after receiving an active cardiac device and/or after decisive experiences such as ICD shock deliveries.

Several reviews have reported on psychopathology among patients with cardiac arrhythmias [62,63,64,65,66,67,68]. Hereby, the prevalence of anxiety disorders and depressive symptoms is estimated to be approximately 20% in ICD patients according to a large meta-analysis from 2011 [6]. These findings were supported by a multicenter trial by Habibovíc et al. in 2020 [69]. However, anxiety could not be connected to the risk for ventricular arrhythmias or mortality [69]. Pedersen et al. presented data on 332 patients, which showed stable psychological functioning in most patients receiving an ICD after 12 months [70]. In addition to that, the same research group evaluated the possible effects of the choice of the ICD system (transvenous ICD or subcutaneous) ICD did not affect the quality of life in ICD patients and was improved by both types of devices [71]. Similar levels were recorded in patients with pacemakers, which was associated with a higher rate of physical and mental fatigue post implantation [72,73]. Interestingly, new evidence evolving by the increasing use of leadless pacemakers as a new technology, shows that leadless pacemakers may be superior regarding quality of life compared to conventional transvenous pacemakers [23]. The elevated levels of psychopathology in cardiac patients support the need to assess and target quality of life in this population [74,75]. In fact, the association of mental health and cardiovascular disease is bidirectional with mental health disorders also negatively influencing cardiovascular morbidity and mortality adding eminent importance to diagnosis and treatment [76,77]. In addition, the results indicate that quality of life goes far beyond screening or diagnosing manifest mental disorders and are therefore worth analyzing and targeting for improvement.

Further attention should also be paid to other possible mediators such as social support. Close relatives like partners or parents of children with implanted devices were examined in recent studies, which revealed that caregiver strain was predictive for the response to LVAD therapy and that parents of children receiving a pacemaker or ICD were at a high risk of developing a post-traumatic stress disorder shortly after implantation but also during follow-up [78]. Consequently, social support for the patients and perhaps also for relatives might be of eminent importance for the therapy outcome [43,44]. Due to the very heterogenic study designs and the lack of reporting in most of the studies, we could not include parameters of social support in the analysis.

The same was true for many data on baseline patient characteristics and outcome parameters. Many studies from our analysis did not include or report data on patient’s functional heart failure status (e.g., NYHA class) or LV function although these factors already have been proven extensively to have a large impact on quality of life [79,80]. However, many of the included trials either showed data on medical outcome (improvement of functional NYHA class, improvement of measured LV function) or parameters of psychological well-being (quality of life, mental diseases). This negatively influences the informative value of the primary studies as well as meta-analyses such as ours as we were not able to investigate whether an improvement of quality life was associated with an improved heart function (e.g., a better LV function) or rather by the device itself. Only few studies from the beginning of ICD therapy for the primary prevention of sudden cardiac death have compared medical therapy to preventive ICD implantation so that valid meta-analysis of controlled effect sizes seem difficult. However, the fact that also ICD implantation, which protects against life-threatening arrhythmias without any effect on cardiac function such as LV-EF, heart rate or rhythm, had a positive effect of quality of life in our study further suggests that there is an effect of cardiac devices itself. CRT-ICD patients who might have benefited from the implantation in terms of heart failure were not included in most studies or if were not analyzed separately. Furthermore, the effects of new technologies such as subcutaneous ICD placement and leadless pacing is too young to be sufficiently analyzed regarding quality of life. Current evidence support a non-inferior or even superior quality of life of these new technologies that have to be further elucidated [23,71]. Possible mediators might be the reduced risk for device related infection and the missing need for abandoned lead extraction. To further improve factors like battery longevity which might have further impact on the increasing use of leadless pacing, several efforts are made so that evidence concerning differences in cardiac as well as psychological endpoints will grow in the next years possibly leading to first-line use of leadless pacing and subcutaneous ICD implantation [81,82]

Our analysis underlines the strong effect cardiac devices may have on patients’ quality of life. Yet, we need more trials assessing measures of quality of life as well as cardiac function and heart failure symptoms at implantation and follow-up to better understand how cardiac device therapy influences patients’ quality of life.

## Data Availability

Raw data can be obtained upon reasonable request from the corresponding author via email (kevin.willy@ukmuenster.de).
